# Focal Infection and Periodontitis: A Narrative Report and New Possible Approaches

**DOI:** 10.1155/2020/8875612

**Published:** 2020-10-29

**Authors:** Jean-Paul Rocca, Carlo Fornaini, Zuomin Wang, Lixin Tan, Elisabetta Merigo

**Affiliations:** ^1^Shijiazhuang 2^nd^ Hospital, Department of Stomatology (South), Gonguong Road 425, Quoxi District, Shijiazhuang, China; ^2^UFRO dontologie, UPR Micoralis, University Côte d'Azur, 24 Avenue des Diables Bleus, 06354 Nice Cedex, France; ^3^Beijing Chaoyang Hospital, Department of Stomatology, Capital Medical University, Beijing, China; ^4^Shijiazhuang 2^nd^ Hospital, Department of Diabetes, Gonguong Road 425, Quoxi District, Shijiazhuang, China

## Abstract

The “focal infection theory” is a historical concept based on the assumption that some infections may cause chronic and acute diseases in different districts of the body. Its great popularity spanned from 1930 to 1950 when, with the aim to remove all the foci of infection, drastic surgical interventions were performed. Periodontitis, a common oral pathology mainly of bacterial origin, is the most evident example of this phenomenon today: in fact, bacteria are able to migrate, develop and cause health problems such as cardiovascular and respiratory diseases, diabetes, and osteoporosis. The aim of this narrative report is to verify the hypothesis of the association between oral infections and systemic diseases by different ways of approach and, at the same time, to propose new kinds of treatment today made possible by technological progress. The analysis of the literature demonstrated a strong relationship between these conditions, which might be explained on the basis of the recent studies on microbiota movement inside the body. Prevention of the oral infections, as well as of the possible systemic implications, may be successfully performed with the help of new technologies, such as probiotics and laser.

## 1. Introduction

The “focal infection theory” is a historical concept theorizing that focal infections may be the cause of many chronic diseases, including systemic and common ones, [1] thus explaining virtually all diseases, including arthritis, atherosclerosis, cancer, and mental illnesses [2, 3].

Focal infection was considered, more than one century ago, as an infection at a specific location able to spread to different districts of the body; in 1931, Graham wrote “by the term “focal infection,” one means a chronic, usually low-grade, infection that develops insidiously and progresses slowly producing local and systemic symptoms”. It is a common primary cause of chronic ill-health, but more often, it acts as a contributing factor in disease conditions primarily due to other causes” [4].

In January 1940, Reimann and Havens published a review and a “critical appraisal” [5] which represent the most influential criticism of the focal infection theory; here, they summarized that “the removal of infectious dental focal infections in the hope of influencing remote or general symptoms of disease must still be regarded as an experimental procedure not devoid of hazard”.

A 1952 editorial [6] in the Journal of the American Medical Association marked the end of the theory era by stating that “many patients with diseases presumably caused by foci of infection have not been relieved of their symptoms by removal of the foci, many patients with these same systemic diseases have no evidence focus of infection, and foci of infection are as common in apparently healthy persons as in those with disease”.

Today, moreover, based on progresses in the understanding of pathogenic mechanisms also with the help of molecular biology, the “focal theory” finds new interest in medicine, as demonstrated by the important number of publications appeared in the literature.

The aim of this narrative report is to give a contribution to understand whether this new interest has to be regarded as a realistic moment in medical science or has to go back to silence; moreover, new possible approaches to the management of these kinds of disease are described.

## 2. Brief History of the Focal Theory

The first report of a possible relationship between oral infections and systemic diseases may be attributed to Hippocrates who described « a patient suffering of rheumatism appearing after a tooth extraction » [7].

The main problem related to this kind of investigation, as focused by Gundrum [8] in 1921, “is the inability to tell whether a given focus is related in a causal way with the symptoms complained of”; moreover, the death and the subsequent revival of the focal theory may be, respectively, related to the antibiotics discovery and to their lower effectiveness due to the evolution of resistance among microbes [9].

An example may be represented by dental focal infections, which are extraoral manifestations caused by oral pathogens. Pathological oral conditions, such as periapical inflammation and periodontitis, can cause bacteremia, and dissemination of oral pathogens to non-oral sites can subsequently cause infections in extraoral tissues and organs. Cardiovascular infections and brain abscesses are the most common of these. The course of such infections can be lethal. In order to improve patient care, a closer collaboration between dental and medical caregivers is necessary [10].

## 3. Commensalism versus Pathogenicity of the Oral Flora

Around 10^14^ bacterial microorganisms are present in the full body (teeth and respiratory, intestinal, and urinary tracts) and around half of them are commensal bacteria. Equilibrium is observed until, for multiple reasons, some species multiplicate while others disappear [11]. The biofilm, for instance, which adheres strongly to the tooth hard surface, is a perfect and accessible model to be studied. This biofilm is embedded in a self-generated extracellular matrix of polysaccharides, and this matrix takes around 85% of the volume of the dental plaque [12].

Moreover, this matrix acts as a diffusion barrier and inhibits binding of some antibiotics. Thus, bacteria organized in biofilms might escape host immune systems [13] and are protected from ecological competition of other microorganisms, thus increasing their pathogenicity [14].

Around 400 to 700 species have been identified, and some of the commensal bacteria may become “opportunistic” species, some of them being strictly considered as pathogens. It becomes possible to hypothesize that they may contribute to local or systemic disorders [15]. On the other hand, the normal microflora is able to colonize permanently and to install a permanent commensal relationship with the host “eating at the same table” (from *Latin* words *cum* = with and *mensa* = table). This microflora participates in multiple beneficial relationships protecting, for instance, from exogenous invasion and infections, by excluding other microorganisms [16]. But, qualitative and quantitative changes are observed during the installation of a biofilm, depending mainly on nutrients (inside a periodontal pocket for instance) and oxygen conditions (anaerobic vs. aerobic or aeroanaerobic or microaerophylic bacteria). It is easy to understand that deeply, in a periodontal pocket, the installation and development of strict anaerobes are favoured. Examples are *Porphyromonas gingivalis* and *Aggregatibacter actinomycetemcommitans*: both these bacteria are able to bypass the epithelial barrier modulating fibroblastic inflammatory response [17]. *Porphyromonas gingivalis*, a Gram-negative strict anaerobe bacterium, is able, for instance, to adhere to the surfaces and may dysregulate the immunologic response at the gingival-epithelial interface. Its virulence factors help to penetrate into the subepithelial interface and represent a key factor that helps the development of periodontal pockets. The connective tissues of the gingiva interact with *P. gingivalis* and its products and activate host inflammatory response [18]. *P. gingivalis*, again, may be considered as a prominent actor in the pathogenesis of periodontitis due to its ability to colonize the oral epithelium and to act as an opportunistic pathogen in the oral environment.

Some questions remain: may*P. gingivalis* have any influence on systemic diseases? What could be the relationship between *P. gingivalis* and other bacterial pathogens of the oral cavity? Is *P. gingivalis* the main species involved in the pathogenicity of periodontitis? May it also play a unique role in focal disease? [19].

The characteristics of a biofilm are the perpetual modifications of the bacteria and the role of each bacterium, including pathogenesis and possible distal problems. Two other examples are needed to try to answer the previous questions.

*Fusobacterium nucleatum* is one of the most common anaerobic Gram-negative bacteria in the oral environment, but it also plays a role in numerous diseases including different forms of periodontitis, endodontic infections and/or respiratory tract infections, rheumatoid arthritis, atherosclerosis, and gastrointestinal problems. It is a good example for the comprehension of focal infection [20]. Its opportunistic “comportment” is observed when linked with prevalence that increases with the severity of the disease (oral or non-oral). *Fusobacterium* (there are 5 subspecies) has been, for a long period, considered as a commensal bacterium implicated in the oral biofilm and more specifically cultivated from dental plaque samples because of its ability to co-aggregate (presence of adhesins) with a lot of different bacterial targets. It binds, for instance, with epithelial and endothelial cells, as well as with fibroblasts [21]. By increasing endothelial permeability, it allows the oral microbiome to penetrate those cells. The “door is open” for mixed infections including extraoral sites [22]. As *Fusobacterium nucleatum* is able to elicit a great variety of host responses, it seems possible to consider that we are speaking about distal (focal) infection.

Among the many bacteria implicated in the pathogenicity of periodontitis and the possible focal dissemination from the oral environment, only some have been explored; in Table 1, the bacterial strains dominant in the oral cavity are reported in order of importance.

More recently, the role of *Aggregatibacter* (ex *Actinobacillus*) *actinomycetemcomitans* has been studied. *A. actinomycetemcomitans* is a facultative anaerobic Gram-negative bacterium considered as an opportunistic pathogen associated with aggressive forms affecting preferentially young patients [24] and able to colonize oral mucosa and produce adhesins allowing adherence to the colonized surfaces (dental hard tissues and oral epithelium). Its relationship with other bacteria in the biofilm is interesting: to survive inside a biofilm *A. actinomycetemcomitans* utilizes products of the metabolism of other species and does not seem disturbed by endogen antibiotics produced by other species (for example, bacteriocins). A characteristic of this bacterium regards the production of exotoxins that affect (stop) the growth of proliferating bacteria [25]. This bacterium produces lysosomal enzymes in the environment and MMPs at the origin of lymphocyte apoptosis.

Why and how does commensalism participate in the regulation of the species in a biofilm? In a periodontal pocket, for instance, a great number of species act as co-residents with perpetual changes in terms of nutrients, competition, production of endotoxins (LPS being linked with the cell walls of Gram-negative bacteria), and many other components. Thus, qualitative and quantitative modifications are observed as described in other environments by Steel et al. [26]. The microbial communities are shaped by interactions among the biofilm population. Some Gram-negative bacteria in other microbial communities, not involved in periodontal problems, are able to inject proteins into neighboring cells. Different mechanisms of intercellular competition exist in this community, and it was demonstrated that, as regards the secretion system of toxins by microorganisms in the “host” gut, the events look like a “fight club” [27]. It seems intellectually acceptable to correlate what happens in a periodontal pocket with endotoxins, exotoxins, enzymes, products of metabolism, pH, oxidative components, and so on. For example, the enzymes of bacterial origin facilitate the penetration of structural barriers destroying proteins that play a role in immune defenses. In fact, homeostasis (mutualistic symbiosis) or dysbiosis will be the result of this cohabitation, depending whether a pathogenic response in the host is observed or not. The microbial warfare plays a role in the intraspecific competition between the host and symbiotic population.

## 4. Endodontics, Periodontology, and Systemic Infections

The interrelationship between periodontal and endodontic disease has always aroused confusion, queries, and controversy: differentiating between a periodontal and an endodontic problem can be difficult, and even if a symptomatic tooth may have pain of periodontal and/or pulpal origin, it is of vital importance to make a correct diagnosis for providing the appropriate treatment [28].

Even if the pathways for the spread of bacteria between pulpal and periodontal tissues are still a subject of controversy today [29], the apical foramen seems to represent the main access route between the pulp and the periodontium, with the participation of all root canal systems: accessory, lateral, and secondary canals, as well as the dentinal tubules through which the bacteria and their products contaminate the medium [30].

In case of competition, as described in the previous paragraph, it is difficult to predict who will get the final supremacy, and for this reason, the main question related to this topic is may the microorganisms in periodontal pockets initiate or aggravate the clinical situation? The answer is that it mainly depends on the dysbiotic ecological changes of oral microbiome, particularly in the depth of the periodontal pockets. Based on the example of *A. actinomycetemcomitans,* we may affirm that bacteria escape the host response because of their ability to invade tissues (in this case oral mucosa), secrete exotoxin, and maintain a serum potential resistance [31]. Moreover, they exhibit a large genetic diversity and may translocate to gingival tissue, then colonizing, developing, and so aggravating the situation.

*A. actinomycetemcomitans* is an early colonizer of the periodontal pocket, and it resists to oxygen and hydrogen peroxide. Subsequently, it may colonize superficial areas of a periodontal pocket, and for this reason, it may be considered as an initiator of the colonisation, also together with some other species; it can be involved in early stages and later in the depth of periodontal pockets, certainly as initiator and also as colonizer, because it causes disruption of the epithelium present in and around the pocket. Its possible implication in focal infection is largely reported in the literature, causing brain abscesses, meningitis, osteomyelitis, endocarditis, and septicemia [32].

## 5. Modern Approach to Focal Theory

Focal theory is also not lacking of controversies today; some authors [20], based on their appreciation on methodological difficulties and “a complete lack of interventional studies,” maintain that the focal infection theory suffers from the absence of evidence, while some others affirm that “data from preclinical animal models and epidemiological studies indicate strong associations between the presence of periodontitis and amplification of a plethora of diseased states, ranging from joint inflammation to cognitive decline” [33, 34].

It seems normal today to consider that the exact contribution of periodontitis to the etiology and the progression of systemic diseases is not strongly established, even if it is also usual to consider periodontitis as having potentially deleterious consequences on the body [35].

An interesting approach to establish a possible relationship between periodontal infections and systemic disease, consisting on epidemiological studies, demonstrated a strong association between chronic periodontitis and cardiovascular disease, respiratory diseases, diabetes, osteoporosis [36], preterm low birth weight [37], and more recently, pancreatic cancer [38], metabolic syndrome [39], chronic kidney disease [40], rheumatoid arthritis [41], and Alzheimer's disease [42].

Recently, some studies have noted a possible association between periodontal diseases and the risk of various cancers, particularly hematological, breast, and prostate [43], while some others have described a positive correlation between periodontal disease and risk of oral, lung, and pancreatic cancers [44]; periodontal disease treatment and prevention might turn out to be important targetable cancer prevention strategies [45].

Severe periodontitis is an important risk factor of gastrointestinal cancer that severely threatens human health, and its control may contribute to early cancer prevention; *P. gingivalis* is probably an important risk factor of gastrointestinal cancer, especially oral cancer, esophageal cancer, colorectal cancer, and pancreatic cancer [46].

Factors involved in the development of a bacterial endocarditis are difficult to define, but a vulnerable surface (i.e., a damaged endocardium) and a high bacterial load in the blood seem to be decisive. The microorganisms involved, in 90% of cases, are staphylococcus, streptococcus, and enterococcus, and as they are part of dental plaque, they could enter the bloodstream causing bacteremia through daily habits such as chewing or tooth brushing [47].

Moreover, *Porphyromonas gingivalis* and *Streptococcus sanguis* [48], *Actinobacillus actinomycetemcomitans*, *Porphyromonas gingivalis*, *Bacteroides forsythus*, *Treponema denticola*, and *Campylobacter rectus* [49], and *Fusobacterium nucleatum-periodonticum-simiae*, *Prevotella intermedia*, *Prevotella nigrescens*, and *Tannerella forsythia* [50] have been found in the thrombi of patients with acute myocardial infarction, even if, with current knowledge, it is not possible to answer the question whether periodontal bacteria become attached to already existing atherosclerotic lesions or these bacteria promote the atherosclerosis and induce instability of plaque [51].

The role of *P. gingivalis* has also been investigated by Olsen et al.; it is able to endure inflammation, as well as to exploit inflammation, for its own sustenance and survival. In addition, *P. gingivalis* can invade periodontal, atherosclerotic, and brain tissue, thereby avoiding immune surveillance and maintaining its viability. By these efforts and through a plethora of other virulence factors, it may act as a keystone organism both in periodontitis and related systemic diseases and other remote body inflammatory pathologies including dementia [52].

Moreover, in a recent human postmortem study, *P. gingivalis*, along with *Treponema denticola*, *Tannerella forsythia*, and their components, was identified in the brain tissue of 10 AD cases within a 12 h postmortem period. The tissues were subjected to immunolabel and immunoblot assays, which confirmed the presence of LPS from P. gingivalis in brain tissues of four AD cases [53].

Regarding the association between oral health and diabetes, most of the studies consider the first as a complication of the second [54–56], and even if the biologic mechanisms behind this relationship are still not completely understood, several studies do show pathologic changes in the gingival vasculature of patients and animals with diabetes, compared to control subjects without DM; examples are basement membrane thickening, angiogenesis, and an increase in osmotic tissue pressure [57, 58].

On the other hand, the chronic inflammatory state induced by untreated periodontitis may contribute to insulin resistance and worsening glycaemic control [59], and while untreated periodontitis poses an inflammatory challenge to the patient, the reduction of periodontal inflammation has potential positive benefits to the patient both locally and systemically [60].

The medical community should be aware of the potential negative effects of periodontal infections on systemic health, first of all by recognizing and treating periodontal infections and, then, by a regular maintenance of oral care. This is the aim of the so-called “Periodontal medicine,” which promotes a strong cooperation between dental and medical professionals; only by a combination of dental and internal medicine implicating a better communication among the stakeholders and an effective team approach in the clinical practice will it be possible to get a real answer to these kinds of diseases [61].

## 6. Future Perspectives

As previously described, molecular biology may contribute to demonstrating the ability of some species of the oral microbiome to induce distal problems today; bacterial microorganisms from the oral cavity are able to directly invade arterial walls or atherosclerotic plaques and can also produce a source of infection (initiation) or aggravate and maintain the previous inflammation. Periodontal pathogens may also enter the circulatory system by destroying the inner epithelium of the periodontal pockets.

Prevention has to become the keyword, and basic periodontal therapy (surfacing and root planning associated with maintenance and hygiene) can reduce the level of systemic inflammatory cytokines and also minimize the risk factors.

The systematic use of antibiotics, in addition to being,as it is largely demonstrated, an effective therapy only in the acute phase of the disease, is under criticism due to the dramatic explosion of the bacteria-resistance increase today [62]; therefore, two new approaches may be considered as a good opportunity for the treatment of periodontal diseases: laser and probiotics.

The antimicrobial capacity of laser devices seems to represent an effective approach in the decontamination of periodontal pockets even if its utilization is still controversial. In fact, while many authors proposed the use of several laser wavelengths able to reduce the inflammatory processes and consequently enable the repair of periodontal tissues, such as Nd:YAG [63], diodes [64], Er:YAG [65], and Er and Cr:YSGG [66], other authors suggested considering laser only as a complement of the conventional treatments (scaling and root planning) [67]; some other authors did not find any differences between the conventional therapy with high power lasers irradiation and the one without it [68].

The role of low energy lasers is different such as in Photobiomodulation (PBM) and Photo Dynamic Therapy (PDT): the difference depends on their use with or without a photosensitizer, and the effect is reached through their photochemical effects.

PBM is a nonthermal process involving endogenous chromophores eliciting photophysical and photochemical events at various biological scales. This process results in beneficial therapeutic outcomes including, but not limited to, the alleviation of pain or inflammation, immunomodulation, and promotion of wound healing and tissue regeneration [69]. Clinical studies have also shown the acceleration of the healing process induced by PBM after mucogingival surgery, as well as after scaling and root planning sessions, and the reduction of gingival inflammation in patients classically more at risk, such as, for example, patients with diabetes mellitus [70].

Over 100 years ago, Oskar Raab discovered the basis of photodynamic therapy (PDT), a therapeutic technique using cytotoxic action for the treatment of infectious diseases and tumors that has been in use since the 1970s [71, 72]. In PDT, the cytotoxic effect is achieved through the local application or systemic administration of photosensitizing agents followed by irradiation of visible light with an appropriate emission spectrum for the absorption spectrum of the photosensitizer in the presence of oxygen [73]. In medicine and dentistry, PDT for antimicrobic purposes is used for different types of applications with different types of photosensitizers and lasers [74]. It has also been used recently with systems based on LED technology [75, 76], and in dentistry, there is growing interest in its application in the treatment of periodontitis and peri-implantitis [77–80], as well as in endodontics [81, 82].

Probiotics, by definition, are viable microorganisms which, when administered in adequate amounts, provide a health benefit to the host. This approach has successfully been used to control intestinal diseases and appears to act through colonisation resistance and/or modulation of the immune system. Likewise, studies are now suggesting that probiotics have the potential to modify the oral microbiota and are being investigated to prevent or treat diseases of the oral cavity, such as dental caries and periodontal diseases, which are associated with a shift in the microbial composition and activity of the biofilm, and the resulting reaction of the host [83].

Two different mechanisms seem to be involved in the control of oral disease:

1. A direct interaction within dental plaque (colonisation resistance): this mechanism could possibly include the disruption of plaque biofilm formation through competition for binding sites on host tissues and other bacteria and through competition for nutrients.
2. Indirect probiotic actions within the oral cavity, including the modulation of both innate and adaptive immune function: within this context, it is possible that lactic acid bacteria interact with immunocompetent cells, such as macrophages and T-cells, leading to an alteration in the production of cytokines and subsequent effects on overall immunity [84].

Many studies involving probiotics as an adjunct to clinical periodontal treatment report a more marked improvement in the clinical status of patients compared to clinical treatment alone, which may represent an important indication for probiotics instead of antibiotics utilization in periodontal treatment to help reduce the overall burden of antibiotic resistance [85, 86].

We know that, today, some bacterial strains may play a protective role in clinical pathological conditions caused by antagonist microorganisms; *S. salivarius* is able to control other different bacteria, responsible of oral dysbiosis, potentially capable of migrating to genitourinary districts: Filkins et al.demonstrated that, in patients affected by cystic fibrosis, the presence of *S. salivarius* in the mouth and in the lungs is much higher in remission times and significantly decreases in acute crisis [87].

Today, several probiotics strains are available to dentists, which may be orally assumed for modifying, in an eubiotic way, oral microbiotic; many studies have confirmed that they may be very effective in controlling oral diseases such as decay and periodontitis.

Among these, K12 strain seems to be very interesting: it derives from *Streptococcus salvarius*, and it was firstly used against *Streptococcus pyogenes* infections and subsequently suggested for a great number of indications; a large amount of research has, in fact, confirmed its efficacy to treat pharyngitis [88], tonsillitis, and otitis both in children and adults [89, 90], in periodontitis [91] and to eliminate halitosis [92]; moreover, a very recent study suggested its successful employment in the treatment of Oral Lichen Planus [93].

It may be so possible to suppose for it a role beyond the oral cavity for modifying, in a eubiotic way, other different organs of the body, even not close to the head-neck district.

## 7. Conclusions

Even if the role of periodontal disease in the generation and maintenance of systemic pathologies seems to be demonstrated by a great number of scientific reports today, the progresses in periodontology and endodontics allow controlling oral infections without removing dental elements, thus assuring a conservative treatment to the patients.

Moreover, the utilization of new technologies and agents may reduce the use of antibiotics, thus reducing the intake of antibiotics and consequently minimizing the risk of bacteria resistance.

## Figures and Tables

**Table 1:** Bacterial strains dominant in the oral cavity where the important role of *Streptococcus* is evident (from the work of Jia et al. [23] modified).

Oral cavity zone	Bacterial strains (in order of numerical presence)						

Hard palate	*Streptococcus*	*Uncl. Pasteurellaceae*	*Veillonella*	*Prevotella*	*Uncl. Pasteurellaceae*		

Tongue dorsum	*Streptococcus*	*Veillonella*	*Prevotella*	*Uncl. Pasteurellaceae*	*Actinomyces*		

Saliva	*Prevotella*	*Streptococcus*	*Veillonella*	*Uncl. Pasteurellaceae*			

Palate tonsils	*Streptococcus*	*Veillonella*	*Prevotella*	*Uncl. Pasteurellaceae*	*Fusobacterium*		

Throat	*Streptococcus*	*Veillonella*	*Prevotella*	*Uncl. Pasteurellaceae*	*Actinomyces*	*Fusobacterium*	*Uncl. Pasteurellaceae*

Buccal mucosa	*Streptococcus*	*Uncl. Pasteurellaceae*	*Gemella*				

Keratinized gingiva	*Streptococcus*	*Uncl. Pasteurellaceae*					

Supragingival plaque	*Streptococcus*	*Capnocytophaga*	*Corynebacterium*	*Uncl. Pasteurellaceae*	*Uncl. Pasteurellaceae*		

Subgingival plaque	*Streptococcus*	*Fusobacterium*	*Capnocytophaga*	*Prevotella*	*Corynebacterium*		

Teeth	*Streptococcus epidermidis*	*Streptococcus*					

Lips	*Streptococcus*	*Candida albicans*					

## Data Availability

The data supporting the conclusions of this paper are available through the articles cited in the reference list.
